# An Introductory Guide to Using Bloomington Drosophila Stock Center and FlyBase for Aging Research

**DOI:** 10.3390/cells13141192

**Published:** 2024-07-14

**Authors:** Xiangzhong Zheng

**Affiliations:** Department of Biology, Indiana University, Bloomington, IN 47401, USA; samzheng@iu.edu

**Keywords:** Bloomington Drosophila Stock Center, aging, lifespan, conserved mechanism, age-related diseases, FlyBase

## Abstract

Studies on numerous species have demonstrated strikingly conserved mechanisms that determine the aging process, from yeasts to worms, flies, zebrafish, mice, and humans. The fruit fly *Drosophila melanogaster* is an excellent model organism for studying the biological basis of normal aging and etiology of age-related diseases. Since its inception in 1967, the Bloomington Drosophila Stock Center (BDSC) has grown into the largest collection of documented *D. melanogaster* strains (currently > 91,000). This paper aims to briefly review conserved mechanisms of aging and provides a guide to help users understand the organization of stock listings on the BDSC website and familiarize themselves with the search functions on BDSC and FlyBase, with an emphasis on using genes in conserved pathways as examples to find stocks for aging studies.

## 1. Introduction

An almost universal phenomenon of life from unicellular organisms to humans is the gradual decline of functions over time, broadly termed aging [1,2]. Cellular senescence is a normal part of aging; however, it is not synonymous with organismal aging in higher-order animals, where organ physiology, crosstalk among tissues [3,4], and host–commensal interactions all play a pivotal role [5,6] in the course of deterioration of physical and physiological functions, along with increased risk of morbidity and mortality. It is a manifestation of complex time-dependent changes at the molecular and cellular levels under variable environments. While chronological age is a commonly used measurement and predictor of age-related functions, the rate of biological aging varies widely among species and within populations [7]. An emerging trend in aging research has highlighted the importance of healthy aging beyond lifespan extension [8]. With the global population aging projected to put an ever-increasing amount of social and economic burden on all countries, extending fitness and function has become the research focus of academic scientists and an investment area for large pharmaceuticals and technology corporations. 

## 2. Conserved Molecular Mechanisms Involved in Aging

### 2.1. The Insulin/Insulin-Like Growth Factor Signaling and mTOR Pathway

Studies from a wide range of species have demonstrated strikingly conserved mechanisms that determine the lifespan and rate of aging from yeasts to the nematode worms, fruit flies, zebrafish, mice, and humans [9,10]. Among them are the insulin/insulin-like growth factor-1 signaling (IIS) and the mechanistic target of rapamycin (mTOR) pathways [11]. These ancient signaling cascades are the main regulators of metabolism and growth. However, a myriad of molecular pathways and biological processes downstream of IIS and TOR are involved in lifespan determination [12,13]. Disruptions of genes encoding positive regulators of IIS and mTOR signaling extend the lifespan. For example, mutations of the insulin receptor *daf-2* almost doubled the lifespan in worms [14], loss of function of insulin receptor (*InR*) and insulin receptor substrate (*IRS*) extended the lifespan in *Drosophila* [12,15,16], and attenuations of InR or insulin-like growth factor 1 (IGF-1) signaling improved longevity in mammals in a tissue and sex-dependent manner [17]. Consistently, loss of the negative regulator FOXO downstream of IIS abolished the effect of IIS mutations on lifespan, whereas overexpression of FOXO delayed aging [18,19,20,21,22]. Furthermore, mounting evidence supports findings from genetic studies: from worms to flies and mammals, caloric restrictions and drugs that target the IIS/mTOR, namely, metformin and rapamycin, extended the lifespan and improved health and age-associated functional decline [23,24,25,26,27,28,29,30,31]. Numerous genes involved in stress responses, cell proliferation, metabolism, and longevity interact with each other, and many of them converge on the IIS/mTOR signaling pathways; reduction in IIS/mTOR activities is associated with fitness and cancer resistance [32,33,34]. Interestingly, analysis of genes involved in the mTOR signaling network from 48 mammalian species also revealed evolutionary features that are unique to long-lived species [31].

mTOR is not only an important regulator of protein metabolism, but it also plays a critical role in autophagy [35]. As a normal part of physiological activities, damaged cellular materials are cleared by the ubiquitin-proteasome and autophagy [36]. During aging, this mechanism is impaired, resulting in a loss of proteostasis that is often associated with neurodegenerative diseases [36,37]. Thus, enhanced autophagy activities have been proposed to have beneficial health effects. Indeed, inhibition of mTOR activates autophagy and improves cellular function and extends lifespan [38]. In support of the central role of autophagy in aging, some diet interventions that enhance longevity require intact autophagy functions [35,39]. 

### 2.2. Mitochondrial Dysfunction

Reactive oxygen species (ROS) are natural byproducts of the oxidative phosphorylation that generates ATP in the mitochondria. Low levels of ROS are important for normal cellular functions, but dysregulated release of ROS causes damage to proteins and cellular compartments [40], including mitochondria. During aging, a decline of the antioxidant system and deterioration of mitochondrial quality control mechanisms such as selective autophagy of mitochondria (mitophagy), further increase oxidative stress, inflammation, and age-dependent diseases [41,42,43,44]. 

Mitochondrial dysfunction is frequently associated with age-dependent metabolic disorders and neurodegenerative diseases [45]. Interestingly, dietary restriction extends lifespan at least partially through activation of the AMP-activated protein kinase (AMPK), which in turn inhibits mTOR activity, promotes mitophagy and improves mitochondrial homeostasis [46,47,48]. Recent studies found that natural compounds such as coumarin and Urolithin A induce mitophagy and extend lifespan in worms [49,50]. Consistently, genetic studies of mitophagy-related genes in model organisms have demonstrated that overexpression of genes that promote mitophagy extends longevity, whereas their loss reduces the lifespan [51].

### 2.3. Inflammation

Innate immunity is the oldest and most conserved aspect of immunity against pathogens in the animal kingdom [52,53]. This acute protective mechanism deteriorates during aging [54], along with more immune cells exiting the cell cycle entering a senescent state known as immunosenescence [12,55]. This weakened immune capacity increases the risk of infection and mortality.

On the other hand, chronic elevation of inflammatory levels without external pathogens is a hallmark of aging [56]. The prominent manifestation of inflammatory status in a myriad of age-dependent diseases, including neurodegenerative diseases, cardio-vascular diseases, metabolic disorders, and autoimmune diseases, has popularized the term “inflammaging” [56,57], which posits that cellular stress is the common trigger of inflammation, and macrophages are central players in the inflammatory events.

### 2.4. Microbiome

The impact of microbiomes, especially the intestinal microbiome, on host physiology, behavior, and “healthspan” (loosely defined as “the period of life spent in good health, free from the chronic diseases and disabilities of aging” [58]) has been well documented [59,60,61,62]. *Drosophila* is an emerging model to study the intersections of diet intervention, microbiota, innate immunity, and aging [12,63]. In addition, gut metabolites play an important role in mediating the anti-aging effect of flavonoids [64].

### 2.5. Circadian Rhythms

One prime example of organism–environmental interactions is the alignment of the daily activities and biological functions with the day:night cycle on earth. The intrinsic circadian clocks orchestrate rhythmic metabolic and physiological processes in virtually all organisms from unicellular cyanobacteria to humans [65]. Disruption of circadian clocks is associated with countless age-dependent diseases, and targeting the circadian clock to extend healthspan has become a new direction in medicine [66,67]. Interestingly, dietary restrictions and the timing of feeding affects the healthspan via the circadian clock [68,69,70,71,72], which is weakened during aging in many organisms [73,74,75]. Autophagy is also intertwined with circadian clocks and sleep [76,77,78,79,80,81,82].

## 3. Age-Associated Diseases and Drug Discovery

Human aging is a systemic decline of physical and physiological functions along with increased risk of diseases, including metabolic syndromes, cardio-vascular diseases, musculoskeletal disease, cancer, immune disorders, neurodegeneration, and cognitive deficits. Injuries from falls, largely due to deterioration of neuro-muscular functions in fine motor control, gait, and balance at old age are among the leading causes of death [83]. With the global population aging, age-related diseases pose an ever-increasing health, social, and economic burden on all countries [84]. To fight Alzheimer’s disease (AD) alone, the United States has implemented a national plan as required by congressional legislation in 2011, with ambitious goals to treat and prevent Alzheimer’s and related dementias. However, two decades of clinical trials of AD drugs have been largely unsuccessful, underscoring the complex etiology of the disease [85]. 

While aging as we currently understand it is irreversible, the rate of aging varies widely among species. It is conceivable and even feasible to slow down biological aging processes [86]. Towards this end, animal models have been used to screen for compounds that extend the lifespan [87]. Pharmaceutical corporations and venture capitals have invested heavily in projects targeting hallmarks of aging and to extend the lifespan [88,89,90], and some promising drug candidates are already in clinical trials.

## 4. *Drosophila* as a Model Organism for Aging Studies

Not surprisingly, genetically tractable organisms such as the baker’s yeast *Saccharomyces cerevisiae*, nematode worm *Caenorhabditis elegans* and fruit fly *Drosophila melanogaster* (hereafter ‘Drosophila’) are popular models of choice for aging studies. Environmental factors aside, genetics is the most significant factor that determines the aging process. Importantly, about 60% of the Drosophila genes have human homologs [91]. Drosophila has complex cellular signaling networks and equivalent organs like those in mammals, a rich repertoire of biological and molecular processes, sophisticated behaviors, and a short reproduction cycle, making it an ideal model to study the mechanism of normal aging and pathological underpinnings of age-related diseases. A large collection of mutants of candidate genes is readily available for drug discovery [92,93,94,95,96,97], validation, and repurposing of FDA-approved compounds for aging studies [29,38].

Since the early 20th century, Drosophila genetics has progressed from discoveries of spontaneous mutant alleles to genome-wide, large-scale generation of allelic mutations, transgenes, and tools for numerous applications for investigations of molecular and cellular processes, development, physiology, neurobiology, behavior, and aging. The rapid expansion of genetic strains necessitates a central repository to maintain the stocks and distribute them to the worldwide research community. Since its inception in 1967, the Bloomington Drosophila Stock Center (BDSC) has grown into the largest collection of live *D. melanogaster* strains with unique genetic compositions (currently >91,000), all of which can be found on the BDSC website listings with stocks by experimental use or feature (category), or by using search functions to locate stocks based on symbols for gene, allele, component, genotype, chromosome, donor, and stock comments. The main goal of this paper is to help users navigate the BDSC website and FlyBase pathway search function to find strains of interest to their studies, with the understanding that genes and pathways involved in aging are selected for the purpose of illustration and discussion. 

## 5. Navigating the BDSC Website

### 5.1. The Overall Organization of the BDSC Website

**Figure 1:**
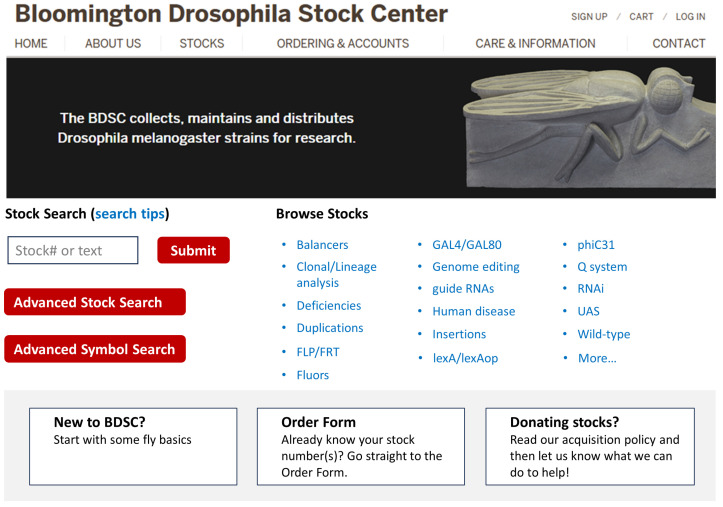
BDSC website homepage layout. The BDSC website (https://bdsc.indiana.edu/) accessed on 11 July 2024) is accessible via any of the major internet browsers on desktop and mobile devices. The “More…” link opens a new page with most pre-compiled stock categories.

The BDSC website (https://bdsc.indiana.edu/) (accessed on 11 July 2024) is designed to make it informative but also easy to navigate. The top ribbon panel of the BDSC website homepage (Figure 1) has six navigation tabs, three of which have a dropdown menu. All website contents can be accessed via this navigation panel. The middle panel consists of two sections: “Stock Search” and “Browse Stocks”, with the latter largely organized by the main components of the stocks: deficiencies, GAL4, lexA, insertions, fluorescent proteins, wild-type etc., along with categories based on stock utilities such as mapping, clonal analysis, genome editing, and so on. The purpose of this layout is to provide quick access to major subcollections without overcrowding the homepage. 

The lower panel consists of three large buttons for easy access to relevant information pages and an Order Form, whether you are new to the BDSC website or have a list of stocks ready to order.

### 5.2. Browsing Tables

When a new stock arrives, BDSC scientists formalize the genetic component symbols and associate various properties in a custom relational database. This strict protocol makes it possible to write queries based on component and stock properties to automatically populate a large number of browsing tables. There are two main entrances to tables with pre-compiled stocks listing genotypes, main components, use comments, and chromosomes. The large subcollections are listed on the homepage (https://bdsc.indiana.edu/) (accessed on 11 July 2024). For example, clicking on the GAL4/GAL80 link on the homepage will open the index page (https://bdsc.indiana.edu/stocks/gal4/index.html) (accessed on 11 July 2024) for all GAL4 and GAL80 stocks. The GAL4/UAS binary system collections are useful for studying the effects of a specific tissue or cell type on lifespan, or the interaction between tissues and cells. About half of the GAL4 transgenes (>8700) carry the intact GAL4 transcription activator that can directly drive the expression of UAS-transgenes. The other half (~7800) carry the split hemi drivers with the GAL4 DNA binding domain or the p65 (or VP16) activation domain, which when combined in the same stock expresses a functional GAL4 driver in the intersection of both hemi-drivers (via leucine zipper pairing). These split hemi-drivers are great for ‘mix-and-match’ studies to explore new expression patterns. In addition, BDSC maintains a set of split combo stocks with a stable combination of transgenes carrying DNA binding domain and activation domain (~3500) donated by the Janelia FlyLight Project (https://www.janelia.org/project-team/flylight) (accessed on 11 July 2024). 

The split-combo webpage can be found on the GAL4 index page (https://bdsc.indiana.edu/stocks/gal4/index.html) (accessed on 11 July 2024). From there, clicking on the split-GAL4 link will open the information page (https://bdsc.indiana.edu/stocks/gal4/split_intro.html) (accessed on 11 July 2024) for all split-hemi, split-combo stocks. On this page, clicking on the split-combo link leads to the split-combo table (https://bdsc.indiana.edu/stocks/gal4/splitcombo_all.html) (accessed on 11 July 2024). Users can filter the column headers using various criteria. Clicking on a link in the table column (Janelia line) will open the image page on the FlyLight website, which details expression patterns in the central nervous system (e.g., https://splitgal4.janelia.org/cgi-bin/view_splitgal4_imagery.cgi?line=SS51118) (accessed on 11 July 2024). Most users search for Janelia line ID based on publications describing its expression pattern (see the FlyLight webpage for details: https://splitgal4.janelia.org/cgi-bin/splitgal4.cgi) (accessed on 11 July 2024).

In an effort to expand the use of the large collection of GAL4 (split-hemi) drivers, BDSC has screened 7300 split-GAL4 lines and characterized the expression pattens of the hemi drivers in the adult intestine (https://bdsc.indiana.edu/stocks/gal4/midgut_splitgal4.html) (accessed on 11 July 2024), which plays an important role in regulating innate immune and inflammatory responses, host–microbiota communication, and aging [98].

Another large collection at BDSC includes the transgenic RNAi stocks generated by the Transgenic RNAi Project (TRiP) [99]. All target genes are listed on this page (https://bdsc.indiana.edu/stocks/rnai/rnai_all.html) (accessed on 11 July 2024), so it is easy to use a filter on the gene column. TRiP is actively developing sgRNA transgenes for gene knockout and gene overexpression. BDSC maintains > 3200 sgRNA transgenes for knockout, and >1800 sgRNA transgenes for target gene expression. The guide RNA webpage (https://bdsc.indiana.edu/stocks/genome_editing/sgrna.html) (accessed on 11 July 2024) provides relevant transgene and vector information and links to the Harvard TRiP project website (https://fgr.hms.harvard.edu/fly-in-vivo-rnai) (accessed on 11 July 2024).

The advent of novel fluorescent probes has greatly expanded the collection of fluorescent transgenes (https://bdsc.indiana.edu/stocks/gfp/index.html) (accessed on 11 July 2024). BDSC has a rich collection of fluorescent reporters that change color or intensity depending on cellular physiological processes, such as changes in cellular pH, calcium dynamics, redox status, membrane potential, ER stress, synaptic connections, and so on. These reporters can be used to investigate age-dependent alterations at the cellular level. This table can be accessed via the homepage: clicking on “Fluors” opens the fluorescent protein index page (https://bdsc.indiana.edu/stocks/gfp/index.html) (accessed on 11 July 2024); from there, clicking on the “Markers, regulator, reporters…” link under the “Fluor-tagged proteins” section opens the fluorescent reporters page (https://bdsc.indiana.edu/stocks/gfp/gfp_mark.html) (accessed on 11 July 2024). Non-fluorescent markers and reporters can be found on the stock index page (https://bdsc.indiana.edu/stocks/index.html) (accessed on 11 July 2024). 

The second way to access the tables of precompiled stocks is the “STOCKS” tab in the top navigation panel (https://bdsc.indiana.edu/stocks/index.html) (accessed on 11 July 2024), which presents most stock categories in an expandable tabular list.

### 5.3. Searches 

To better utilize the search tools on the BDSC website, it is important to know how genetic information is curated at BDSC. A stock genotype is comprised of text strings called components, i.e., alleles, insertions, balancers, and so on. For each component, the associated genes and nature of the allele, tag, descriptive comment such as gene expression, genomic location (map), feature or use of the component, and the original publication that characterizes the component are parsed into various database tables. Donor, publication (if multiple components of a specific stock are used for a particular experiment), descriptive comments about the purpose or use, and synonyms of the stock are associated with the stock number in the BDSC database. Note that visible markers are added to the genotype for transgenes (the text string in the genotype between “{“ and “=”, e.g., PBac{y[+mDint2] w[+mC]=UAS-hAKT1.HA}VK00037), but not associated with components in the database. These relational associations form the basis of the search functions described in the sections below.

#### 5.3.1. Stock Search

The Stock Search interface can be accessed at https://bdsc.indiana.edu/Home/Search (accessed on 11 July 2024), from the homepage (https://bdsc.indiana.edu/) (accessed on 11 July 2024), or from a link on the other two search pages. This is a quick search using simple terms in a single search box to fetch stock data that match any of the following terms:

- stock number

- gene symbol (note that BDSC uses FlyBase [100] symbols for genes)

- component symbols

- text string of gene symbol, component symbol, genotypes, stock comments, donor names.

Note that a wildcard Boolean (*) is added to both ends of the search term, and if multiple terms (separated by space) are entered, an “AND” operator is automatically used in the query, and the order of terms is ignored. This Stock Search often retrieves a long list of stocks. While it takes effort to wade through the list, a desirable set of stocks can be obtained by checking the results and refining the terms in a few trials. It also has the added benefit of finding related stocks of which users may be unaware. 

If a single stock number is entered into the Stock Search box, it automatically retrieves that stock, instead of searching the whole database for text strings containing that number. A combination of numbers separated by a space will be treated as combination of text strings. Likewise, a combination of a number and a text string (separated by space) will be treated as text strings with an “AND” operator. It will search gene symbols, component symbols, genotypes, comments, and donor names, but not stock numbers. 

Because the Stock Search uses text strings to search many text fields in the database, the query is designed to ignore certain invalid inputs. For example, if only a single letter is entered into the Stock Search box, such as N (gene symbol for Notch), p (gene symbol for pink), it is automatically rejected because numerous records contain such single letters. Likewise, entering an invalid term (e.g., a random text string) that is not contained in the database will return no record, but if it is combined with another text string that is contained in the database, the results of the valid search term are returned while the invalid term is ignored (otherwise, the invalid search term will nullify the valid query). 

In addition, Greek symbols need to be spelled out in the Stock Search term: i.e., type “betaTub85D”, instead of “βTub85D”; “Delta1” instead of “Δ1”. It is worth noting that FlyBase has changed the gene symbol for Dl to “Delta” to avoid confusion with the gene symbol for dorsal (dl). Thus the search term “Delta” brings out alleles and transgenes for the gene Delta, but it also fetches other records with the text string Delta/delta in gene symbols such as deltaTry (δTry), allele and transgene symbols GLaz[Delta2], P{UAS-Psn.527.deltaE9}2, and so on. 

#### 5.3.2. Advanced Stock Search

**Figure 2:**
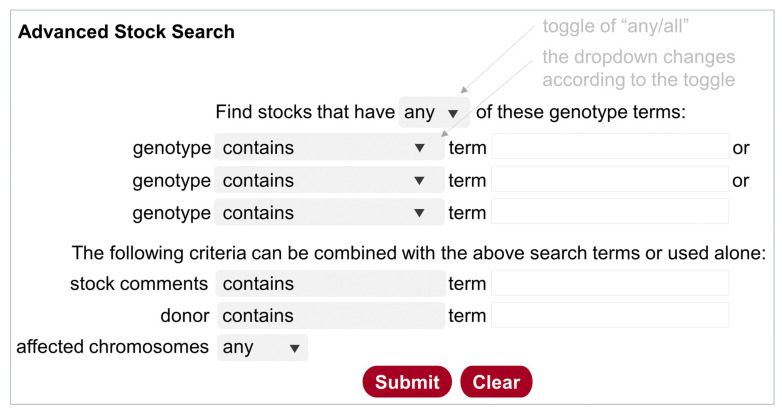
Illustration of the Advanced Stock Search. Clicking on the top toggle of “any/or” changes the query design accordingly, i.e., the Operator “or” is used for “any”, and the Operator “and” is used for “all”. The gray boxes contain a dropdown list that is shown by clicking on the triangle.

While the Stock Search described above can take multiple search terms, they are not defined to specific fields in the database. The Advanced Stock Search (Figure 2) is designed specifically for stocks (genotype, donor, stock comment, chromosomes affected), but not for components. It can be accessed at https://bdsc.indiana.edu/Home/AdvancedSearch (accessed on 11 July 2024), from the homepage (https://bdsc.indiana.edu/) (accessed on 11 July 2024), or from a link on the other two search pages. Users can add three search terms (text strings) for genotypes. The toggle “any/all” at the top can be used to include or exclude stocks in the hit list. Furthermore, text strings from the stock comments or donor names can be used either alone or in combination with genotype terms. It is good practice to use “any” and “contain” to retrieve a more inclusive list first, then use a combination of restrictions to narrow down the list. This search function is particularly useful for finding stocks with combinations of components on specified chromosomes. Many users also find it convenient to use the donor’s name to find all stocks from that donor. 

#### 5.3.3. Advanced Symbol Search

**Figure 3:**
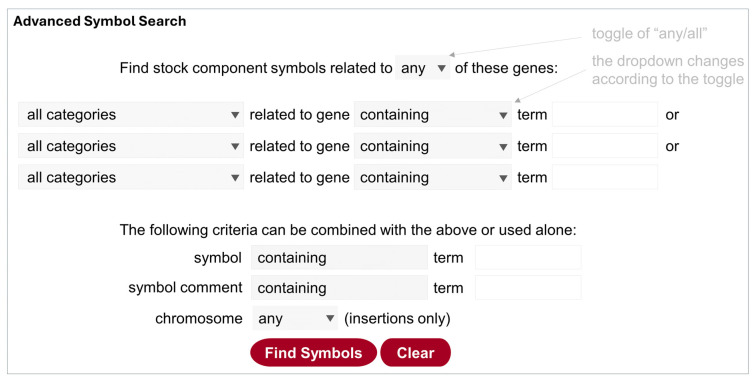
Illustration of the Advanced Symbol Search. Clicking on the top toggle of “any/or” changes the query design accordingly, i.e., the Operator “or” is used for “any”, and the Operator “and” is used for “all”. The gray boxes contain a dropdown list that is shown by clicking on the triangle. Category terms are further explained in Table 1.

This search function is best suited to find a component with specific features (such as gene trap, noncoding RNA etc.) (Figure 3). It can be accessed at https://bdsc.indiana.edu/Home/AdvancedComponentSearch (accessed on 11 July 2024), from the homepage, or via a link at the bottom of the other two search pages. Here the “symbol” refers to a stock component, i.e., parts of a genotype that comply with the FlyBase nomenclature, for example, InR[E19] (allele), PBac{UAS-hAKT1.HA}VK00037 (transgenic insertion), Df(2R)Atg7[d77] (deficiency), and CyO (balancer). Because visible markers for transgenes are only added to genotypes, they should be excluded in the component symbol search. 

Because all components are associated with gene information such as the nature of the allele/insertion, users can easily narrow the hit list to a specific type of allele/insertion for the gene of interest, by selecting the dropdown menu (the default is “all categories”; see Table 1 for explanations). Switching the toggle at the top (“any/all”) changes the dropdown menu for genes and symbols, so different set of terms can be used to either expand or narrow the search further. In addition, the lower panel can be used together with the upper panel or alone to search text strings in symbols or comments. For insertions, users can specify which chromosome is affected. 

Unlike that of the Stock Search and Advanced Stock Search, the search results of the Advanced Symbol Search are components that can be selected to retrieve stocks that carry them.

### 5.4. Useful Tips for Searches

a. All search items are treated as case-insensitive. 

b. FlyBase symbols are used in the BDSC database and on the website. For example, the microtubule-associated protein tau, implicated in Alzheimer’s disease, has the Human Genome Organization Gene Nomenclature Committee (HGNC) gene symbol ‘MAPT’. BDSC follows FlyBase nomenclature of non-*melanogaster* gene with the four-letter prefix of the genus\species abbreviation: Hsap\MAPT. Because it is commonly known as “tau” in the literature, BDSC adds the common name to MAPT in the comment field (e.g., expresses human MAPT (tau) under the control of UAS) to help users find all transgenes carrying MAPT, either using the search term ‘MAPT’ or ‘tau’. 

c. Stock comments and component comments are usually different. In most cases, information related to the use of the strain and notes on balancers and markers are attached to a stock, while descriptive comments related to the nature of the construct and allele are associated with individual components of a genotype. In Stock Search, both comment fields are used, whereas the Advanced Stock Search and Advanced Symbol Search use their specific comment fields. 

d. For users unfamiliar with Drosophila nomenclature, these interspecies mining tools are very useful to find fly ortholog/paralogs: Gene2Function [101] (https://www.gene2function.org/search/web/) (accessed on 11 July 2024) and BioLitMine [102] (https://www.flyrnai.org/tools/biolitmine/web/) (accessed on 11 July 2024). 

### 5.5. Sharing Information between BDSC and FlyBase 

To understand the smooth transition between BDSC and FlyBase websites, it will help to know how stock data are processed into the BDSC database and how such data are validated against FlyBase, a comprehensive database of genetic and molecular data for *D. melanogaster* and other *Drosophila* species [100,103]. Donor-provided genotypes and publications will be first checked to see if FlyBase has curated symbols from that reference, including transgenic insertions, alleles, aberrations (duplication, deficiency, inversion). For components that already have FlyBase curations, BDSC adds the corresponding FlyBase ID (FBid) for genes and components, so they are linked to FlyBase when the new stocks are published on the BDSC website. If the donor-provided reference has not been curated by FlyBase, BDSC will ask FlyBase to curate the paper; if the components have not been published, BDSC will send personal communications to FlyBase with additional donor-provided information such as the nature of the allele, transgene generation, expression, and chromosomal location. These new components usually get their corresponding FlyBase ID at the next public release of FlyBase webpages.

**Figure 4:**
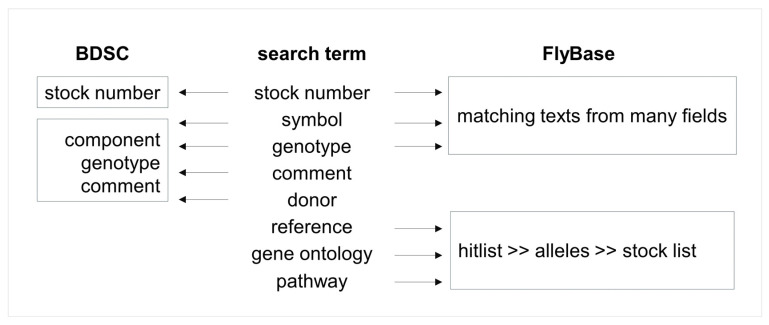
Flow chart for using BDSC or FlyBase to find stocks. Use BDSC for stock numbers since it has the most current information about genotype and stock availability. Use FlyBase for reference, gene ontology, and pathway searches. Symbols (gene, component) on the BDSC webpage are linked to FlyBase, which provides vast genetic and molecular data, linkouts, and references for further reading.

BDSC also updates FlyBase with new stock information in every release cycle. At the same time, genotypes of all stocks are parsed into individual components to compare with FlyBase symbols, and the ones without matching FlyBase records are updated in the BDSC database. All known components are associated with alleles and genes in the FlyBase database, and their corresponding FlyBase IDs are checked against the BDSC database and the missing IDs are backloaded. Thanks to this tight association of BDSC stock components with the FlyBase database, clicking on the component on the BDSC stock page will open a FlyBase report page with detailed information about the component; users can also take advantage of the vast curation information on FlyBase [100,103] to find stocks. Figure 4 provides a simple decision flow on when to use BDSC or FlyBase for relevant information. As discussed in multiple sections below, a list of genes retrieved through a FlyBase Pathways search can be easily converted into symbols, which then can be converted to stocks with a button click.

### 5.6. Ordering Stocks

Stocks found through the Stock Search or Advanced Stock Search can be easily added to the Cart by clicking on the “Add to Cart” button next to the genotype in the search results. Components found through the Advanced Symbol Search need to be selected (using a check box next to the symbol), before clicking on the “Find Stock” button at the bottom of the search results page. If a user already has a list of stock numbers from other sources, use the “Order Form” on the BDSC homepage. The checkout process is similar to many e-Commerce websites that require account information and agreement to terms of stock use. It should be noted that stocks added to the Cart when a user is not logged in will be lost by clearing the browser data (in local storage); stocks added to the cart while logged in are not affected (i.e., these data are stored on the web server). 

## 6. Finding Stocks for Studying Conserved Mechanisms 

### 6.1. IIS and mTOR

The IIS and mTOR signaling pathways have been extensively studied, so finding stocks for these well-known genes is easy with the Advanced Symbol Search. Nonetheless, it is difficult to devise a search model that fits all needs. For example, the gene symbol “Tor” has been used by the Drosophila community for a long time before its recent change to “mTor” to better distinguish it from the “tor” (gene symbol for torso). There are 42 gene symbols containing the text string “tor” in the BDSC database so it is not a good term to search. “mTor” is contained in eight gene symbols (including Lamtor, Samtor). Thus, using the “exactly matching” term “mTor” is the best way to obtain a clean list (22 symbols currently). Note that the numbers given in this paper are for illustration purposes. They may change over time due to stock additions/losses or gene symbol changes. 

**Figure 5:**
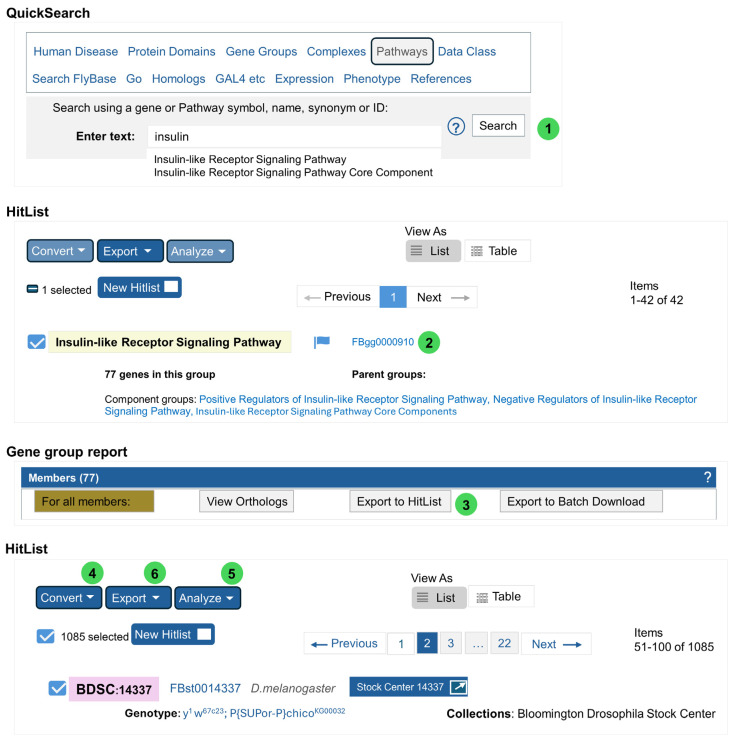
Using a FlyBase Pathways search to find stocks. (1). On the FlyBase homepage (https://flybase.org/) (accessed on 11 July 2024), select “Pathways” and enter “insulin” in the search box, then select “insulin-like Receptor Signaling Pathway” from the dropdown. (2). Click on the gene group link (FBgg0000910) to open the Gene group report page. (3). In the “Members” section, click on the “Export to HitList” to fetch alleles and stocks. (4). Select “Stocks” from the dropdown menu. (5). Select “Collection” and then “Bloomington Drosophila Stock Center” in the popup window. (6). Select “Batch Download” to export the list (stock number, genotype). Note that the numbers in this illustration and other sections are based on FB2024_03, released 25 June 2024.

To find a comprehensive list of genes in the IIS and mTOR signaling pathways, users are encouraged to use a Pathways search on FlyBase: for example, the hit list for insulin signaling (77 genes) (FB2024_03, released on 25 June 2024) can be converted to 1085 BDSC stocks (Figure 5). Clicking on the “Stock Center…” link will open the stock report page on the BDSC website. The hit list can also be exported, and stock numbers can be uploaded to the Order Form on the BDSC homepage. Further mining of the gene group [100] data (e.g., Insulin-like Receptor Signaling Pathway https://flybase.org/reports/FBgg0000910) (accessed on 11 July 2024) helps narrow down the list to those core components, negative and positive regulators, and those with physical interactions. 

### 6.2. Autophagy

The effect of the nutrient-sensing pathway on aging is in part mediated by autophagy. Many autophagy-related genes (ATGs) are highly conserved from yeasts to mammals [104]. Genetic studies further demonstrate the role of autophagy in aging: Atg7 mutant flies are hypersensitive to nutrients and are short-lived [105]. On the other hand, overexpression of Atg genes extends the lifespan [106,107,108]. A Stock Search of “autophagy” returns two stocks, both with a comment “autophagy marker” or “marker for autophagy” in the component comments. A Stock Search of “Atg” returns 279 stocks, including a lot of deficiencies that cover the Atg gene and non-relevant stocks with a comment on the translation start codon ATG, or a comment on fragment sequence such as CATGATGAAATAACA. 

The Advanced Stock Search has limited use to retrieve all stocks related to Atg gene because many of them do not have the gene symbol in the genotype, e.g., the TRiP RNAi or gRNA stocks. In addition, a collection of the Rab GTPase transgenes that contains “Rab40-GAL4.ATG” are included in the results. 

An Advanced Symbol Search for a gene that contains “Atg” returns 227 components, including deficiencies that cover the Atg gene region. After selecting the components by using the check box next to the component on the search results page, clicking on the “Find Stocks” button at the bottom of the page returns all relevant stocks. Excluding the deficiency returns 144 stocks. In comparison, a search of FlyBase data class “gene group” with “Autophagy” returns 20 autophagy-related genes (all symbols start with Atg). This hit list can be converted to stocks (225 stocks), which can be further filtered by the “Analyze” function to filter on “collection” (see the example in Figure 5): the Bloomington collection holds 135 stocks. These two lists have 134 stocks in common. Nine stocks are found only in the BDSC list; among them, two are GAL4 enhancer trap insertions between Atg1 and Sap130, five are transgenes carrying human ATG genes, and the remaining two are newly added stocks after the last FlyBase release. Because all gene symbols in the Autophagy gene group starts with “Atg”, users can refine the search as any component related to gene “starting with” “Atg”, which then excludes the five human Atg gene symbols starting with “Hsap\ATG”.

Mitophagy is a process that recycles damaged mitochondria by autophagy. The FlyBase gene ontology term “mitophagy” includes six genes (three overlap with autophagy-related genes and the other three genes involved in mitochondrial homeostasis). This hit list can be converted to 42 BDSC stocks (with 16 overlaps with the “autophagy” hit list). 

The term “mitophagy” has been essentially dissociated with autophagy in the description of alleles and genetic tools. As such, a search of “mitophagy” on the BDSC website returns five stocks carrying transgenes expressing fluorescent proteins fused with the mitochondrial localization peptide, with a comment that they are used as “mitophagy reporters” (mito-QC, matrix-QC); none of them is present in the search results for “autophagy”. This example illustrates the value of curating the “use” information for genetic reagents. Indeed, a large number of BDSC webpages are organized into this type of “use” category. For example, the same five stocks can be found through browsing the fluorescent reporter page (https://bdsc.indiana.edu/stocks/gfp/gfp_mark.html) (accessed on 11 July 2024): filtering the “Reporter/marker” column in the table on this page with “mitophagy” will narrow down the list to five mitophagy reporter stocks. 

### 6.3. Inflammation

Drosophila lacks adaptive immunity, but its innate immune system shares similarities with that of vertebrates [109] and it has become an excellent model to study host–pathogen interactions, innate immune memory, and inflammatory disease [110]. In addition to the recruitment of antimicrobial peptides to infection sites, IL-like receptor Toll and Nuclear factor-κB (NF-κB) cascades are activated to trigger an inflammatory response. NF-kB is a family of transcription factors (such as Dif, dl, Rel) involved in different processes of the immune and inflammatory responses. Interestingly, the IIS activity is correlated with NF-kB activity [111]. Constitutive activation of the NF-kB and Imd signaling pathways is associated with neurodegeneration and shortened lifespan in Drosophila and mice [56,111]; conversely, suppression of Toll/NF-kB or Imd signaling improves the lifespan [111,112,113]. These data suggest that NF-kB may serve as a therapeutic target to improve age-related diseases and extend the lifespan.

A Stock Search for “inflammation” on the BDSC website returns nothing, because this term is not contained in gene symbol, genotype, or stock comments. Similarly, searching “inflammation” on FlyBase returns 396 records in Disease Ontology but only six genes, which include the gene immune deficiency (imd). However, searching “immune” returns 851 alleles/insertions (associated with 1059 stocks, of which 725 are in BDSC); and 445 records in Gene Ontology; among them, “immune response” alone has 358 annotated genes (associated with 5280 stocks, including 2986 BDSC stocks). These large stock lists are comprehensive, but they are not helpful in picking specific stocks. Here the FlyBase curation of gene groups becomes handy. For example, within the Gene Ontology term “immune response”, there are 15 records in the gene groups, including the antimicrobial peptides group (99 stocks), Imd signaling pathway core components (339 stocks), positive (231 stocks) and negative (481 stocks) regulators of the Imd pathway, and so on. These smaller subsets make it easier for users to select specific stocks. Furthermore, most Drosophila researchers search stocks for their gene of interest, so the gene-centric curation provides sufficient information to find specific stocks. For example, searching the gene “imd” on FlyBase returns nine BDSC stocks, the same result as when using the Advanced Symbol Search on BDSC (excluding the Deficiency stocks). 

### 6.4. Oxidative Stress

Excessive ROS in metabolically active cells such as neurons are implicated in age-dependent diseases like Alzheimer’s and Parkinson’s diseases. Genes encoding ROS scavenger proteins are well-studied in Drosophila, and gene symbols are unequivocal for these well-known enzymes; thus, a quick Stock Search for Sod, Cat, and Gst will return relevant stocks. To find regulatory genes important for oxidative stress responses, using the FlyBase gene ontology (GO) search for “oxidative stress” will pull out a comprehensive list of GO terms, such as “cellular response to oxidative stress” and “response to oxidative stress”. Annotated genes in each GO term can be converted to stocks as illustrated in Figure 5. 

### 6.5. Microbiome and External Stressors 

While a well-characterized wild-type strain is suitable to study the effect of microbiome composition on host physiology, the large collection of mutant strains at BDSC is a great resource for studies on gene–microbiota interaction and its impact on aging. Although large-scale applications for genome-wide screening of longevity mutants are not readily available for most laboratories, creative approaches have been used to speed up the identification of candidate genes. For example, unlike the traditional QTL mapping, extreme QTL mapping [114] can be employed for selection-based phenotyping of pools to recover mutants with extreme trait values, such as resistance to stressors and pathogens. Indeed, the Drosophila Synthetic Population Reference (DSPR) [115] panel has been successfully used to uncover the genomic loci associated with caffeine and xenobiotic resistance [116]. It is not unconceivable that many large collections of BDSC stocks can also be used to select extreme phenotypes under external stressors that are known to have strong impact on aging. Because most BDSC stocks have characterized sequence features, whole-genome sequencing of DNA pools of phenotyped individuals can help trace back to the genetic component responsible for the extreme phenotype. The readily available sequence information for the *Drosophila melanogaster* Genetic Reference Panel (DGRP) [117] and DSPR collections makes them extremely useful for quantitative genetic analysis of the effect of gene–microbiome interactions on healthspan. These two collections can be found via the “Wild-type” link on the BDSC home page.

To test the effect of gene expression in the midgut on aging, a set of 424 split GAL4 drivers [118] that are expressed in at least one of five major midgut regions can be used with the UAS-transgene of choice to express or knock down genes in specific cell types and midgut regions (https://bdsc.indiana.edu/stocks/gal4/midgut_celltypes.html) (accessed on 11 July 2024); among them, 88 split GAL4 drivers have limited expression in the enteroendocrine cells (https://bdsc.indiana.edu/stocks/gal4/midgut_EEs.html) (accessed on 11 July 2024).

### 6.6. Circadian Rhythms

BDSC has a collection of transgenes and mutants of circadian clock genes. One can use the combination of Stock Search and Advanced Symbol Search to find all stocks related to core circadian clock genes. For example, using “circadian” in a Stock Search returns 20 stocks, which include the ten stocks from the “rhythm” search. All of them have the text string “circadian” or “rhythm” in component or stock comments. A Stock Search for “clock” returns nothing, because “clock” is not in the gene symbol, genotypes, or comments. 

Although there is currently no curation of circadian rhythms (clock) page on the BDSC website, FlyBase has a very nice Gene Ontology record for circadian rhythms (169 genes), which includes all 121 genes in the GO term “circadian behavior”. The hit list can be converted to 5389 stocks (3585 in the BDSC collection). This huge discrepancy between the BDSC search and FlyBase data mining is due to the fact that GO is a gene-centric curation of any genes reported to be involved in circadian rhythm regulation, and the conversion of the hit list to stocks is a query of the FlyBase database to fetch all stocks carrying any alleles of those genes; in contrast, BDSC component or stock comments are entered into the database when the stock is added to the collection, and usually it is prompted by donor-provided information (e.g., ‘this stock can be used for circadian rhythm experiments’). A good example is ebony (e): there are 1216 stocks on FlyBase (782 in the BDSC collection). Since the discovery of ebony mutations in 1923, ebony alleles had been extensively incorporated into stocks as morphological markers, before the role of ebony in circadian rhythms was reported [119]. Thus, data-mining results often need manual curation. For example, stock 1678 Ki[1] p[p] by[1] cu[1] red[1] e[1]/TM3, Sb[1] Ser[1] is clearly not a good pick as a circadian clock mutant strain, even though it has the e[1] allele. 

The most effective way to retrieve these stocks is to use the “Advanced Symbol Search” on the BDSC website. It is recommended to use “exactly matching” gene symbols. One search of “per” or “tim” or “Clk” returns 93 stocks, another search of “cyc” or “Pdp1” or “vri” returns 99 stocks; and another search for “exactly matching” dco or starting with CkII or starting with Pdf returns 154 stocks. The same method can be applied to other genes involved in circadian rhythms: cwo, tws, cry, sgg, nmo, and so on.

## 7. Screening for Novel Mechanisms of Aging

As alluded to above, genome-wide screening for lifespan mutants is often cost-prohibitive and time-consuming. Nonetheless, one can take advantage of the large collection of stocks at BDSC for genetic screens of enhancer/suppressor- and tissue-specific effects on aging [120]. For example, a short-lived strain with X chromosome-linked alleles (or autosomal dominant alleles) can be combined with a suitable GAL4 to cross with a panel of UAS-RNAi lines and screen for lifespan extension. Extreme QTL mapping [114] can be applied to screen for natural variations that interact with known lifespan shortening alleles. BDSC has two complementary collections of wild-type strains with genome sequence data: DGRP and DSPR. The DGRP collection is wild-caught iso-female inbred lines from Raleigh, NC, USA. It has been used in >125 studies (see aggregated phenotype data at https://dgrpool.epfl.ch/) (accessed on 11 July 2024). The DSPR collection was established by interbreeding eight wild-type strains caught worldwide. Comprehensive information about these stocks and analytical tools are available at the DSPR website (https://wfitch.bio.uci.edu/~dspr/index.html) (accessed on 11 July 2024). In a similar vein, short-lived alleles can be used to screen for compounds that can reverse the deleterious effects of the known genetic mutation on lifespan. 

During aging, the ability to climb decreases [121]. This simple negative geotaxis behavior can be used for genetic screens to identify super-climbers (those who maintain neuromuscular functions during aging). By using a semi-automated climbing assay for physical vigor and neuromuscular coordination, cross-sectional surveys can help identify candidate strains in a manageable time window. 

## 8. Concluding Remarks

While the main focus of this paper is on aging, the genetic tools available for manipulating the Drosophila genome are among the most sophisticated in any multicellular organism, making it a versatile model organism to study fundamental biological mechanisms relevant to human health. As the largest Drosophila repository in the world, BDSC facilitates biomedical research by maintaining and distributing large collections of mutants and transgenic stocks, and by providing information and tools to identify stocks for specific projects. A synergistic collaboration between BDSC and FlyBase helps researchers find stocks and related information on both websites. The BDSC website provides a comprehensive list of tips for searches (https://bdsc.indiana.edu/search/search_tips.html) (accessed on 11 July 2024). As always, BDSC scientists are available to help users find any stocks. Users are encouraged to send feedback to BDSC (https://bdsc.indiana.edu/contact/index.html) (accessed on 11 July 2024). Questions and input help BDSC improve data dissemination and webpage construction.

## Figures and Tables

**Table 1:** Dropdown menu description of gene–component symbol associations.

Dropdown Item	Description
all categories	No filter for any category
allele	Classical gene alleles, insertions associated with altered genomic DNA
antibody fragment against	Gene product targeted by antibody expressed from the transgene
coding	Insertions carrying coding sequence for the gene
coding and regulatory	Insertions carrying both coding and regulatory sequences for the gene
deficiency or putative deficiency	Deficiencies that delete or may delete the gene
gene trap	Insertion sequences may be transcribed with the gene
guideRNA	Guide RNAs for the gene
inserted or swapped in	Insertions in the gene
noncoding RNA	Insertions carrying a noncoding RNA gene
potential misexpression	Insertions carrying UAS sequences that might direct expression of nearby genes
protein binding site (e.g., FRT or loxP site)	Transgenes harboring non-regulatory DNA sequences (e.g., recombinase target sites)
protein trap	Insertion sequences (usually reporter) may be incorporated into the protein
regulatory or putative regulatory	Insertions carrying regulatory or putative regulatory sequences from the gene
RNA sponge	Insertions carrying RNA for sequestering the gene transcript
RNAi	Insertions carrying sequences for RNAi against the gene
wildtype allele	Wild-type alleles of the gene
Zn finger nuclease	Insertions carrying Zn finger nuclease that targets the gene

## Data Availability

The data presented in this paper are query results of BDSC and FlyBase database. There are no archived datasets.
